# Central precocious puberty in a 3 year-old girl with Phenylketonuria: a rare association?

**DOI:** 10.1186/1472-6823-14-38

**Published:** 2014-04-28

**Authors:** Laura Lucaccioni, Bernd C Schwahn, Malcolm Donaldson, Claudio Giacomozzi

**Affiliations:** 1 Paediatric Unit, Department of Medical and Surgical Sciences for the Children and Adults, University of Modena & Reggio Emilia, via del Pozzo n. 71, 41125 Modena, Italy; 2 Metabolic Unit, Royal Hospital for Sick Children, Dalnair St, Yorkhill, G3 8SJ Glasgow, UK; 3 Paediatric Endocrinology Unit, Royal Hospital for Sick Children, Dalnair St, Yorkhill, G3 8SJ Glasgow, UK; 4 Dipartimento di Medicina Pediatrica, Bambino Gesù Children's Hospital, Piazza S. Onofiro 4, 00165 Rome, Italy

**Keywords:** Precocious puberty, Phenylketonuria, Hyperphenylalaninaemia, Gonadotropin-realising hormone agonist treatment

## Abstract

**Background:**

Central precocious puberty (CPP) and phenylketonuria (PKU) are two rare conditions, the latter being the rarer. To date, only one case featuring both these conditions has been reported, and hyperphenylalaninemia was assumed triggering CPP.

**Case presentation:**

We present a 3.2 years old girl referred with a 12 months history of breast and pubic hair development, and vaginal discharge. Hyperphenylalaninemia had been identified by newborn screening and PKU subsequently confirmed by plasma amino acid and genetic analysis. Early dietary control of plasma phenylalanine had been excellent afterwards, resulting in phenylalanine concentrations consistently within the recommended range. Clinical scenario, hormonal assessment and imaging were in keeping with true idiopathic central precocious puberty. Treatment with long lasting gonadotropin-releasing hormone analogue led to regression of secondary sexual characteristics.

**Conclusion:**

We describe for the first time CPP in a girl affected with PKU but with persistently well controlled blood phenylalanine concentrations. This finding is in contrast to a previous report which suggested persistently high phenylalaninemia levels as potential trigger for CPP in PKU patients. Our report, together with the lack of evidence in published cohort studies of children with PKU, strongly suggests this rare association is coincidental and independent of the presence of severe hyperphenylalaninemia.

## Background

True Central Precocious puberty (TCPP), also called GnRH-dependent precocious puberty, can be defined as true puberty, mediated by the hypothalamus, with the onset of secondary sexual characteristics before the age of eight in girls and nine in boys [1-4]. The estimated incidence of TCPP is 1:5.000-10.000 throughout the world [2] with a female:male ratio of between 3:1 and 23:1 [3]. It is less common than, and should be differentiated from, other forms of sexual precocity such as premature thelarche and exaggerated adrenarche. Early activation of the hypothalamic-pituitary-gonadal axis (HPGA) in TCPP is mostly idiopathic, especially in girls [1-4]. However TCPP may be secondarily related to brain tumors (hamartoma especially), brain infections, congenital brain defects, cranial irradiation, insults and injuries to the brain or spinal cord (including cerebral palsy, hydrocephalus and brain ischemia) [4]. Moreover TCPP has been widely described in association with learning disability syndromes, including Angelman, Kabuki make-up syndrome and other genetic conditions in which mental retardation is a key clinical feature [5-8]. Phenylketonuria (PKU) is an autosomal recessive disease caused by a deficient activity of Phenylalanine Hydroxylase (PAH), the enzyme which metabolizes Phenylalanine (Phe) to Tyrosine (Tyr). Deficiency of PAH results in tyrosine deficiency, hyperphenylalaninemia (HP) and accumulation of Phe in other body fluids [9]. If left untreated or not appropriately controlled, patients may develop mental retardation, microcephaly and epilepsy due to the high sensitivity of the immature brain to pathological Phe concentrations. Insufficiently treated patients can also have behavioural problems and some may suffer from psychiatric illnesses [10]. To our knowledge only one girl affected by HP has been described in the medical literature with associated TCPP [11]. In their paper the authors highlight the poor control of serum Phe level due to their patient’s inadequate adherence to the low Phe diet and hypothesized that chronically elevated Phe concentrations were the trigger of TCPP. Since then, no other data supporting or rejecting this conjecture have been reported. We present the case of a three-year-old girl affected by severe PAH deficiency who was diagnosed with TCPP, and have compared her to the single case previously reported, pointing out differences and similarities.

## Case presentation

**Figure 1:**
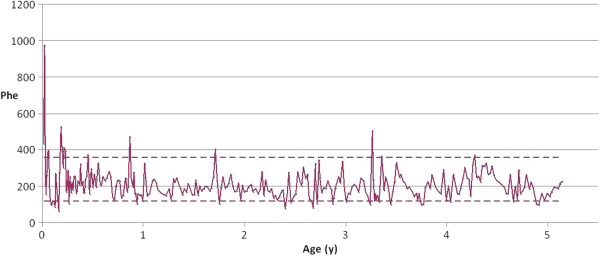
**Patient serum Phe concentration from birth.** The area within dotted line represents the treatment target range. Phe concentration is in μmol/l.

A girl was referred to our endocrinology outpatient clinic at 3.2 years with a one year history of increasing breast enlargement followed by the development of pubic hair, body odour and brown vaginal discharge. She had been born at 38 weeks gestation by caesarean section due to maternal pre-eclampsia, birth weight 2.7 kg (−0.72 SD)*.* Her family history was negative for developmental anomalies. Newborn screening revealed HP with a Phe level of 435 μmol/L on day 5 and she was started on dietary treatment from day 9 of life once severe isolated hyperphenylalaninaemia had been confirmed with plasma amino acid analysis (975 μmol/L). Bi-directional sequence analysis of the PAH gene identified two heterozygote mutations: p.Phe39Leu which is associated with a variably severe phenotype, as well as a second well-characterised null mutation, p.Pro281Leu. Her phenylalanine tolerance remained around 200 mg per day throughout the first 2.5 years of life. She showed normal neurodevelopment but for transitory mild speech delay. From infancy to the time of referral the girl underwent weekly Phe measurements with median (range) values of 194 μmol/L (63–527) and thus almost exclusively within the target range of 120-360 micromol/l for children from 0 to 5 years of age in the UK (Figure 1). On examination in the endocrine clinic the girl was tall in comparison to her parents’ height, 0.8 SDS according to normal reference [12], while her mid parental height (MPH) was −1.2 SDS, with a height corrected for MPH of 2.0 SDS. Pubertal staging according to Tanner’s method [13] was assessed at B4, P2, A1.

## Results

Bone age was advanced by over one year at 4.62 years using the TW2 (RUS) method [14] leading to a predicted final height below the cut-off of −2.5 SDS. An LHRH stimulation test was arranged and showed a pattern which was diagnostic for TCPP (Table 1). Pelvic ultrasound scan showed a bulky uterus of 4.75 cm length with a fundo-cervical-ratio of 1.17 and endometrial thickness of 5.7 mm. Ovaries were 1.70 and 0.78 ml in volume, both within the normal volume range for age although large follicles were evident. Brain and pituitary MRI showed a normal pituitary gland with a mild convexity presentation compatible with the HPGA activation, but no other organic causes for TCPP were detected. Treatment with the GnRH agonist (Triptorelin) was started at the dosage of 11.25 mg every twelve weeks. After nine months, because of persistent breast enlargement and increased height velocity, the interval between injections was reduced to ten weeks. Subsequently the girl showed regression of breast development and a decrease in height velocity. Now aged 4.6 years the patient remains on treatment, showing reduced height velocity and regression of secondary sexual characteristics (Table 1). Throughout treatment her dietary management had been adjusted. Phenylalanine tolerance remains elevated at around 350 mg per day on GnRH agonist treatment and Phe concentrations have been maintained within the target range with median (range) values of 194 μmol/L (63–527).

**Table 1:** Clinical and biochemical features in a girl with phenylketonuria presenting with sexual precocity aged 3 years

**Age**	**3.2 yr**	**3.9 yr**	**4.2 yr**	**4.6 yr**
Height, cm	98.8 (0.8)	107.5 (1.5)	109.7 (1.4)	111 (1.2)
Weight, Kg	16.1 (0.8)	18.54 (1.1)	18.76 (0.9)	19.44 (0.8)
BMI, Kg/m^2^	16.43 (0.4)	16.0 (0.2)	15.6 (0.0)	15.7 (0.1)
MPH, cm	156.0 (−1.2)			
Ht corrected for MPH, SDS	2.0	2.7	2.6	2.4
Bone age, yr	4.62	-	5.37	-
Ht velocity, cm/y	14.3 (5.3)	10.6 (2.9)	7.1 (0.1)	4.3 (−2.2)
Pubertal status	B_4_,P_2_,A_1_	B_2_,P_2_,A_1_	B_1–2_,P_1_, A_1_	B_1–2_,P_1_, A_1_
Basal/Peak LH, U/l	1.9/33.1			
Basal/Peak FSH, U/l	2.4/6.5			
Triptorelin dose	11.25 mg/12 weeks	11.25 mg/10 weeks	11.25 mg/10 weeks	11.25 mg/10 weeks

SDS is presented in brackets. Height velocity assessed according to Tanner et al. [13].

## Conclusions

Phenylketonuria was first described by Asbjørn Følling in 1934 as one of the most common inherited metabolic disorders. The overall incidence of PKU in Europe and North America is 1:10.000-15.000 live births [15]. It occurs, however, more frequently in certain areas such as Turkey (1:3.500-1:5.000), Ireland (1:4.500) or the West of Scotland (1:7.500) [16]. Patients can be classified on the basis of residual enzyme activity as judged by peak blood Phe levels or, better, long-term phenylalanine tolerance. Phe-restricted diets with amino acid and micronutrient supplementation are highly effective in preventing irreversible brain damage and allow for a normal physical and cognitive development [10]. The recommendations for acceptable safe blood Phe concentrations are to some extent age specific [17]. Our patient has a severe enzyme deficiency with a low phenylalanine tolerance, increasing the risk for neurocognitive anomalies. Her parents achieved an excellent dietary control of Phe concentrations with subsequent normal neurodevelopment. To our knowledge, there is only one previous case reported in the literature [11] describing TCPP in a Turkish girl aged 7.5 years with severe PAH deficiency. While most countries represented in the medical literature have universal neonatal screening programs in place to avoid symptomatic PKU, classical PKU was diagnosed late in this patient at 2.5 years of age, indicating that she had been exposed to elevated Phe concentrations from birth. Moreover, satisfactory Phe concentrations could not be achieved in the girl because of poor adherence to dietary recommendations. The authors assumed that persistent poor dietary control could have prompted TCPP due to a premature activation of the HPGA by a toxic effect on the brain. Concerning this latter conjecture, there are currently insufficient data in the literature as to the incidence of TCPP in untreated or insufficiently treated individuals with PAH deficiency. However, expert opinion corroborated by a poll of the metabolic listserv “metab-l” suggests that this is an extremely rare occurrence. Our patient is the first case where idiopathic TCPP occurred under conditions of perfect dietary control. Since encountering TCPP in our patient we have performed a literature search to identify studies that describe the growth pattern of patients with PKU, but none of them focused on the timing of pubertal onset. Recent studies of larger cohorts of individuals on PKU diets have identified an increased prevalence of obesity and overweight towards adolescence in female subjects, with no specific records about precocious or early pubertal progression [17,18]. Lack of evidence for different timing of pubertal onset in PKU patients compared with the general population corroborates our observation, and strongly supports the notion of a co-incidental association between PKU and TCPP. We can not entirely exclude, however, that significant anomalies in the onset and tempo of puberty in PKU patients might have been overlooked by previous population studies.

We conclude that TCPP is a rare coincidental event in children with PKU and can occur independently by the persistently high phenylalanine concentrations.

## Consent

Written informed consent was obtained according to the parents of the patient for publication of this Case report and any accompanying images. A copy of the written consent is available for review by the Editor of this journal.

## Abbreviations

CPP: Central precocious puberty; PKU: Phenylketonuria; TCPP: True central precocious puberty; HPGA: Hypothalamic-pituitary-gonadal axis; PAH: Phenylalanine hydroxylase; Phe: Phenylalanine; Tyr: Tyrosine; HP: hyperphenylalaninemia; LH: Luteinizing hormone; FSH: Follicle stimulating hormone; Cr: Creatinine.

## Competing interests

The authors declare they have no competing interests.

## Authors’ contributions

LL reviewed the case note and performed the first draft of the manuscript. CG and MD followed the patient from and Endocrine Point of view, while BS from a metabolic point of view. CG supervised the draft of the manuscript and reviewed it with MD and BS. All authors read and approve the final manuscript.

## Pre-publication history

The pre-publication history for this paper can be accessed here:

http://www.biomedcentral.com/1472-6823/14/38/prepub
